# *Enterococcus faecium* WB2000 Inhibits Biofilm Formation by Oral Cariogenic Streptococci

**DOI:** 10.1155/2011/834151

**Published:** 2011-10-26

**Authors:** Nao Suzuki, Masahiro Yoneda, Yuko Hatano, Tomoyuki Iwamoto, Yosuke Masuo, Takao Hirofuji

**Affiliations:** Section of General Dentistry, Department of General Dentistry, Fukuoka Dental College, 2-15-1 Tamura, Sawara-ku, Fukuoka 801-0193, Japan

## Abstract

This study investigated the inhibitory effect of probiotic *Enterococcus faecium* WB2000 on biofilm formation by cariogenic streptococci. The ability of *E. faecium* WB2000 and JCM5804 and *Enterococcus faecalis* JCM5803 to inhibit biofilm formation by seven laboratory oral streptococcal strains and 13 clinical mutans streptococcal strains was assayed. The *Enterococcal* strains inhibited biofilm formation in dual cultures with the mutans streptococcal strains *Streptococcus mutans* Xc and *Streptococcus sobrinus* JCM5176 (*P* < 0.05), but not with the noncariogenic streptococcal strains. *Enterococcus faecium* WB2000 inhibited biofilm formation by 90.0% (9/10) of the clinical *S. mutans* strains and 100% (3/3) of the clinical *S. sobrinus* strains. After culturing, the pH did not differ between single and dual cultures. The viable counts of floating mutans streptococci were lower in dual cultures with *E. faecium* WB2000 than in single cultures. *Enterococcus faecium* WB2000 acted as a probiotic bacterial inhibitor of cariogenic streptococcal biofilm formation.

## 1. Introduction

Dental caries is a very common chronic disease arising from the interplay among the oral flora, teeth, and dietary factors. The major etiological players are the two *α*-hemolytic “mutans group” streptococci: *Streptococcus mutans *and *Streptococcus sobrinus* [1, 2]. These bacteria adhere tenaciously to glucan-coated surfaces, produce large amounts of extracellular polysaccharides, and are highly acidogenic and acid tolerant [3, 4]. The colonization of dental plaque by *S. mutans *and *S. sobrinus *plays a causative role in dental caries. Sugar metabolism is central to the behavior of mutans streptococci, and sucrose, the most cariogenic dietary carbohydrate, is used to produce the extracellular polysaccharides that form the biofilm matrix, which aids in the association of mutans streptococci with dental plaque. Once the mutans streptococci biofilm becomes part of the dental plaque, the acidic byproducts of sugar fermentation dissolve tooth enamel, eventually resulting in dental caries [4]. Dental plaque contains numerous genetically distinct types of bacteria that live in close juxtaposition on host surfaces [5]. The “viridans group” streptococci, such as *Streptococcus sanguinis*, *Streptococcus oralis*, *Streptococcus mitis*, and *Streptococcus gordonii*, are noncariogenic streptococci. Together with *Actinomyces *spp., they play an important role as initial colonizers of the tooth surface in the formation of dental plaque [6]. 

Probiotic bacteria, defined as live microorganisms that confer a health benefit on the host when administered in adequate amounts (FAO/WHO 2001), are thought to play a role in the maintenance of oral health [7]. Studies examining the effects of oral probiotics demonstrated that the consumption of products containing *Lactobacillus rhamnosus *or *Lactobacillus reuteri* reduces the number of mutans streptococci in the oral cavity [8, 9]. The use of probiotic tablets containing *Bifidobacterium* spp. normalized the microbiota in patients with periodontitis and gingivitis, as compared with a control group [10]. *Lactobacillus salivarius* taken orally improved the periodontal health of healthy volunteers, particularly smokers, and reduced the numbers of periodontal bacteria in subgingival plaque [11, 12]. The administration of *L. salivarius *WB21 to patients with oral malodor reduced the malodor and bleeding on probing from the periodontal pocket [13]. Therefore, the probiotic bacteria used in the human oral cavity are mainly *Lactobacillus *spp. and *Bifidobacterium* spp., whereas *Enterococcus* strains have been used in human systemic health, especially as probiotic supplements to counter gastrointestinal diseases [14, 15], and they are widely used as veterinary feed supplements [16, 17]. 

Enterococci are facultative anaerobic, Gram-positive cocci that form a part of the normal gastrointestinal tract flora in animals and humans. They are also frequently found in fermented food that is consumed raw, such as cheese and meat, as well as in vegetables and olives [18]. The genus *Enterococcus *includes at least 23 species, two of which, *E. faecalis *and *E. faecium*, account for more than 95% of the clinically important isolates. Traditionally, they are regarded as low-grade pathogens, but they have emerged as an increasingly important cause of nosocomial infections [19]. They are rarely found in the healthy human oral cavity, yet *E. faecalis *appears to occur frequently, albeit in low numbers, in primary root canal infections, especially in teeth with coronal leakage [20]. The clinical use of *E. faecalis *and *E. faecium *during food fermentation and as probiotics demands careful safety evaluation [21]. Recently, Kumada et al. reported that *E. faecium *129 BIO 3B (Biofermin Pharmaceutical, Kobe, Japan) inhibited biofilm formation in dual cultures with *S. mutans*, *S. sobrinus*, and *S. sanguinis in vitro* [22]. To examine the suitability of using probiotic *E. faecium *WB2000, which was isolated from the feces of a healthy human and is contained in the Japanese gastrointestinal agent Strong Wakamoto (Wakamoto Pharmaceutical, Tokyo, Japan), to maintain oral health, this study examined the inhibition of biofilm formation by viridans group streptococci or cariogenic mutans streptococci in dual cultures with *E. faecium *WB2000 using 96-well microtiter plate assays. 

## 2. Methods and Materials

### 2.1. Bacterial Strains and Culture Conditions

*Enterococcus faecium *WB2000 previously classified as *Streptococcus faecalis* [23], which was provided by Wakamoto Pharmaceutical, as well as *E. faecium *JCM5804, *E. faecalis *JCM5803, *S. mutans *JCM5705 and Xc, *S. sobrinus *JCM5176, *S. gordonii *DL1 (*Challis*), *S. sanguinis *American Type Culture Collection (ATCC) 10556, *S. oralis *ATCC 10557, *S. mitis *ATCC 903, 10 clinical strains of *S. mutans* (SMW01, SMW03, SMW08, SMW09, SMW10, SMW11, SMW13, SMW15, SMW22, and SMF01), and three clinical strains of *S. sobrinus* (SSW07, SSW14, and SSW24) were examined. The 13 clinical isolates of mutans streptococci, which have the ability to form, biofilms, were isolated from the saliva of 35 healthy adult volunteers (23 males and 12 females, aged 25–61 years, with a mean age of 38.5 ± 9.8 years [±SD]) using selective medium (CRT bacteria; Ivoclar Vivadent AG, Schaan, Principality of Liechtenstein) and the polymerase chain reaction. The volunteers consisted of workers employed by Wakamoto Pharmaceutical Co. (Tokyo, Japan) and coauthors at the Fukuoka Dental College. Permission for this study was obtained from the Ethics Committee for Clinical Research at Wakamoto Pharmaceutical Co. and at the Fukuoka Dental College (approval number, 2010-02 and 163, resp.). After growth to stationary phase, the bacteria were suspended into skim milk and stored as 1-mL aliquots in sterile tubes at −80°C. 

The ability of the clinical mutans streptococcal isolates to form a biofilm in a 24-well (flat-bottom) microtiter plate (Sumitomo Bakelite Co., Tokyo, Japan) was determined as follows. A single colony was cultured in 1 mL of GAM both with 2.0% sucrose for 24 h, and the supernatant was discarded. The well was rinsed three times with sterile distilled water (d-water), air-dried, and stained with 0.1% crystal violet solution for 15 min. After staining, the well was rinsed with d-water to remove excess dye and air-dried. The presence of a biofilm mass was confirmed by visual assessment.

### 2.2. Human Saliva Preparation

Whole saliva samples were collected from three healthy human participants (mean age, 31.7 ± 5.5 years) by their chewing paraffin gum. The mixed saliva was centrifuged and 10 000 ×g for 20 min at 4°C, and the supernatant was incubated at 56°C for 30 min to inactivate degradative enzymes. The samples were sterilized using a sterile membrane filter (pore size, 0.22 *μ*m; Millipore, Billerica, MA, USA) and used immediately for the biofilm formation assay.

### 2.3. Biofilm Formation Assay in 96-Well Microtiter Plates

Biofilm formation by each strain was assayed using a described method [22]. To start the biofilm formation assay, precultures of each bacterium stored at −80°C were grown in 10 mL of brain heart infusion (BHI) medium (Difco Laboratories, Detroit, MI, USA) for 24 h at 37°C to full growth. To evaluate biofilm formation in cocultures of *Enterococcus* spp. and oral *Streptococcus* spp., 20 *μ*L of an enterococcal cell suspension and 20 *μ*L of the other bacterial cell suspension were mixed in a well of a 96-well (flat-bottom) microtiter plate (Nunc A/S, Roskilde, Denmark), along with 160 *μ*L of tryptic soy broth (without dextrose, supplemented with 0.25% sucrose; TSBS; Wako Pure Chemical Industries, Osaka, Japan), after coating the plates with whole saliva for 30 min at 37°C. To evaluate biofilm formation by single cultures, 20 *μ*L of bacterial cell suspension and 180 *μ*L of TSBS were added to each well. The plates were incubated at 37°C for 16 h under anaerobic conditions, and the liquid medium was removed. The wells were rinsed a second time with d-water, air-dried, and stained with 0.25% safranin for 15 min. After staining, the plates were rinsed with d-water to remove excess dye and then air-dried. The biofilm mass was dissolved with ethanol, and the stained biofilm was quantified by measuring the absorbance at 492 nm using a microplate reader (Sunrise Rainbow Thermo; Tecan Group, Männedorf, Switzerland). The pH of the supernatant was determined before and after culturing for 16 h.

### 2.4. Determination of the Viable Bacterial Count

To evaluate the change in bacterial cell numbers in the biofilm formation assay, diluted supernatant of the reaction mixture was cultured on GAM (Nissui, Tokyo, Japan) agar plates containing 2.0% sucrose for enterococci or on GAM agar plates containing 2.0% sucrose and 0.2 units mL^−1^ bacitracin (Sigma, St. Louis, MO, USA) for mutans group streptococci. After a 24 to 48 h incubation at 37°C under anaerobic conditions, the viable bacteria were counted.

### 2.5. Reproducibility and Statistical Analysis

Each assay was performed in at least three wells per plate, and at least three independent replicates were performed. The differences in biofilm formation by the laboratory streptococcal strains between single cultures and dual cultures with the enterococcal strains were analyzed using the Kruskal-Wallis test. For paired comparisons of biofilm formation using clinical mutans streptococci and *E. faecium *WB2000, the Mann-Whitney test was used. Differences at the 0.05 level were considered statistically significant.

## 3. Results

### 3.1. Effect of *E. faecium* and *E. faecalis* on Streptococcal Biofilms

**Figure 1:**
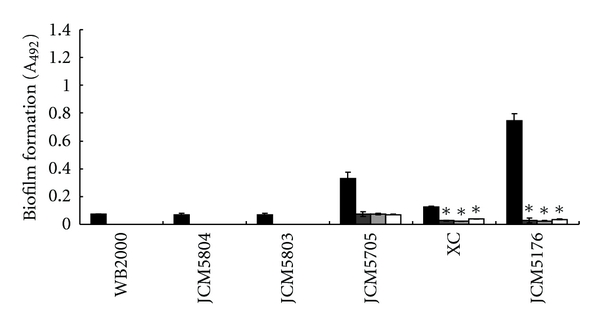
Inhibitory effects of *E. faecium* WB2000 and JCM5804 and *E. faecalis *JCM5803 on biofilm formation by laboratory mutans group streptococci. WB2000, *E. faecium *WB2000; JCM5804, *E. faecium *JCM5804; JCM5803, *E. faecalis *JCM5803; JCM5705, *S. mutans *JCM5705; Xc, *S. mutans *Xc; JCM5176, *S. sobrinus *JCM5176. Black bars: single culture; dark gray bars: dual culture with *E. faecium *WB2000; light gray bars: dual culture with *E. faecium *JCM5804; white bars: dual culture with *E. faecalis *JCM5803. *P < 0.05 versus single culture.

The biofilm formation analyses of single cultures showed that *E. faecium *WB2000 and JCM5804 and *E. faecalis *JCM5803 produced little biofilm (Figure 1, lines 1–3). The viridans group streptococci also made little or no biofilm, except for *S. oralis* ATCC 10557 (data not shown). The *Enterococcus *strains did not inhibit biofilm formation by *S. oralis,* and a mixture of the other viridans group strains and *Enterococcus *strains did not make a biofilm. *S. mutans *JCM5705 and Xc and *S. sobrinus *JCM5176 made biofilms in single cultures (Figure 1, lanes 4–6), and every *Enterococcus *strain inhibited this biofilm formation. In particular, the biofilm formation by *S. mutans* Xc and *S. sobrinus *JCM5176 was significantly inhibited (*P* < 0.05). 

**Figure 2:**
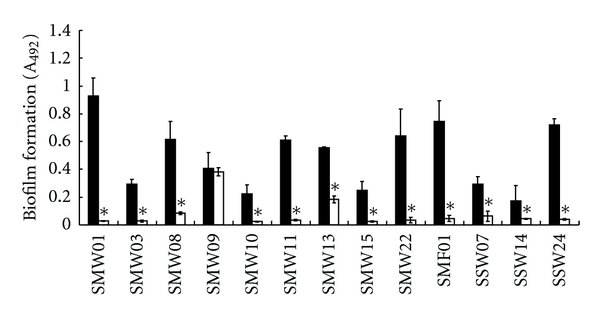
Inhibitory effects of *E. faecium *WB2000 on biofilm formation by clinical mutans group streptococci. SMWs and SMF, clinical *S. mutans *strains; SSWs, clinical *S. sobrinus *strains. Black bars: single culture; white bars: dual culture with *E. faecium *WB2000. *P < 0.05 versus single culture.

Subsequently, 10 clinical *S. mutans* strains and three clinical *S. sobrinus *strains were examined. The single cultures resulted in various amounts of biofilm formation (Figure 2, lanes 1–13). The biofilm formation was reduced significantly in dual cultures of nine *S. mutans *(90.0%) and three *S. sobrinus *(100%) clinical strains with *E. faecium *WB2000. *E. faecium *WB2000 and JCM5804 and *E. faecalis *JCM 5803 had similar effects on reducing biofilm formation. The pH in the single cultures of *S. mutans *and *S. sobrinus *ranged from 4.6 to 4.8, and the pH in the single culture of *E. faecium *WB2000 was 4.9. The pH of the dual cultures of mutans streptococci with *E. faecium *WB2000 was 4.8–4.9.

### 3.2. Viable Bacterial Count

**Figure 3:**
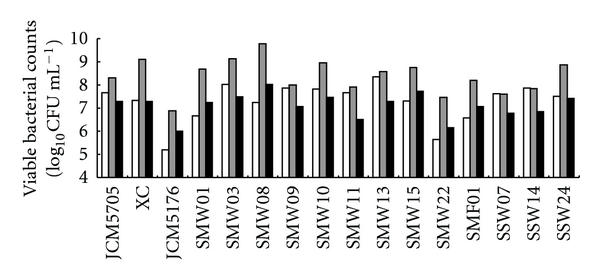
Viable bacterial counts of strains of mutans group streptococci in single cultures at 0 and 16 h and in dual cultures with *E. faecium *WB2000 at 16 h (log_10_CFU mL^−1^). JCM5705, *S. mutans *JCM5705; Xc, *S. mutans *Xc; JCM5176, *S. sobrinus *JCM5176; SMWs and SMF, clinical *S. mutans *strains; SSWs, clinical *S. sobrinus *strains.

**Figure 4:**
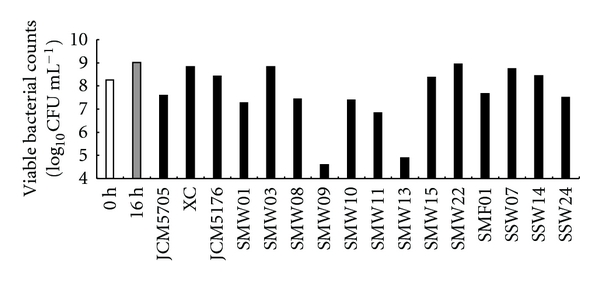
Viable bacterial counts of *E. faecium *WB2000 in single cultures at 0 and 16 h and in dual cultures with strains of mutans group streptococci at 16 h (log_10_CFU mL^−1^).

A viable count was performed to reveal whether the decrease in streptococcal biofilm formation was caused by the death of bacterial cells or by another factor such as inhibition of cell adhesion. Figures 3 and 4 show the viable bacterial counts of mutans group streptococci and *E. faecium *WB2000 in single cultures at 0 and 16 h and in dual cultures at 16 h. After culturing, the numbers of all of the bacterial strains in the dual cultures were lower than those in single cultures. The numbers of nine of the mutans streptococci in dual cultures with *E. faecium *WB2000 at 16 h were reduced in comparison with those at 0 h. The pattern of the increase or decrease in the numbers of streptococci and enterococci varied. In dual cultures of *S. mutans *SMW09 and *E. faecium *WB2000, in which biofilm formation was not inhibited, the number of *E. faecium *WB2000 was markedly reduced (Figure 4, lane 9).

## 4. Discussion

This study evaluated the capacity of *E. faecium *WB2000 to inhibit biofilm formation by oral viridans group and mutans group streptococci. This organism markedly inhibited biofilm formation by mutans streptococci, as reported previously [22], but did not inhibit biofilm formation by *S. oralis* in the viridans group. The other three viridans group streptococci formed little or no biofilm in single cultures, and the combinations of viridans group streptococci with *Enterococcus *spp. resulted in equal or reduced total biofilm mass compared with the single enterococcal cultures. Standar et al. [24] reported that *S. mitis *failed to form biofilm structures in similar *in vitro *assays, but that the combination of *S. mitis* with *S. mutans *resulted in an increased total biofilm mass compared with cultures of *S. mutans *alone. Bacterial interactions are very important for biofilm formation. To confirm the specific inhibition of cariogenic streptococci by *E. faecium *WB2000, a future study should examine multiple bacteria. 

In general, *E. faecium *WB2000 inhibited biofilm formation by the clinical mutans group streptococci, except for *S. mutans *SMW09, which was an exception to the probiotic effect of *E. faecium *WB2000. Nevertheless, planktonic numbers of mutans streptococcal strains cultured with *E. faecium *WB2000 were lower than those in single culture at 16 h. These results indicate that *E. faecium *WB2000 in dual cultures possessed bacteriostatic or bactericidal activity against mutans streptococcal strains. The viable cell numbers of *E. faecium *WB2000 in dual cultures also varied. In particular, the number of *E. faecium *WB2000 cultured with *S. mutans *SMW09 decreased markedly by 16 h. Overall, single cultures of the clinical strains produced various amounts of biofilm, and the increase or decrease in the number of bacteria in a reaction was strain dependent. These phenomena depended on differences in characteristics among the clinical strains and intercellular reactions. Deng et al. [25] examined the effects of *S. mutans* on *E. faecalis *biofilm formation using eight clinical *E. faecalis *strains with the ability to form biofilms and found differences among the strains. 

A study of lactobacilli suggested that overgrowth and superinfection by a probiotic bacterium in the oral cavity are not a concern, although the stability of the exogenous bacteria in the oral cavity is unclear. In our previous study [13], the number of *L. salivarius *WB21 in saliva reached a peak on day 15 and did not increase further until day 29. Shimauchi et al. [11] reported that the populations of *L. salivarius *in saliva specimens from healthy volunteers in both test and placebo groups tended to decrease during the intervention period. Lactic acid bacteria commonly found in the oral cavity are *Lactobacillus *spp. and *Bifidobacterium* spp., whereas *Enterococcus* spp. are primarily in the large intestine and rarely in the oral cavity. The stability of *E. faecium *WB2000 and maintenance of its activity in a complex oral microflora should be examined in the future. 

*Enterococcus *spp. are traditionally regarded as low-grade pathogens, but have been recovered from the oral cavity, mainly from the dental plaque of individuals with underlying diseases in whom opportunistic infections have occurred [26]. We examined the application of *E. faecium *WB2000 in oral health maintenance because this strain is harmless and has rarely been recovered from the oral cavity. For more than 50 years, *E. faecium *WB2000 has been used in traditional Japanese medicine (Strong Wakamoto^®^) to treat gastrointestinal discomfort, and *E. faecium *has been used more often than *E. faecalis* to treat gastrointestinal trouble in humans. Compared with *E. faecalis*, *E. faecium *appears to pose a lower risk for use in foods because its strains generally possess fewer recognized virulence determinants [27] probably due to the presence of pheromone-responsive plasmids. A well-documented example of the safety of exogenous enterococci is *E. faecium *strain SF68, which has been used in pharmaceutical preparations to treat diarrhea [15, 28]. This organism does not have any enterococcal virulence factors and is not able to adhere to vascular epithelial cells or endocardial cells [29]. No case of infection with probiotic *E. faecium *strains has ever been reported in humans. Although additional investigations are required to clarify the mechanism by which *E. faecium *inhibits biofilm formation by cariogenic mutans streptococci and to determine the stability of the *E. faecium* population the present study may provide valuable data regarding the use of an *E. faecium *strain as local therapy for oral infections.
